# Satisfaction with cognitive remediation therapy: its effects on implementation and outcomes using the cognitive remediation satisfaction scale

**DOI:** 10.1038/s41537-023-00390-9

**Published:** 2023-09-30

**Authors:** Joanne Evans, Rose Tinch-Taylor, Emese Csipke, Matteo Cella, Andrew Pickles, Paul McCrone, Dominic Stringer, Abigail Oliver, Clare Reeder, Max Birchwood, David Fowler, Kathryn Greenwood, Sonia Johnson, Jesus Perez, Rosa Ritunnano, Andrew Thompson, Rachel Upthegrove, Jon Wilson, Alex Kenny, Iris Isok, Eileen M. Joyce, Til Wykes

**Affiliations:** 1 Institute of Psychiatry, Psychology and Neuroscience, King’s College London, London, UK; 2 South London and Maudsley NHS Foundation Trust, London, UK; 3 School of Health Sciences, University of Greenwich, London, UK; 4 UCL Queen Square Institute of Neurology, University College London, London, UK; 5 Warwick Medical School, University of Warwick, Coventry, UK; 6 School of Psychology, University of Sussex, Brighton, UK; 7 Faculty of Brain Sciences, University College London, London, UK; 8 Cambridgeshire and Peterborough NHS Foundation Trust, Cambridge, UK; 9 School of Psychology, University of Birmingham, Birmingham, UK; 10 Norfolk and Suffolk NHS Foundation Trust, Norwich, UK; 11 Patient Advisory Board, King’s College London, London, UK

**Keywords:** Diseases, Schizophrenia

## Abstract

Cognitive Remediation (CR) improves cognition and functioning but is implemented in a variety of ways (independent, group and one-to-one). There is no information on whether service users find these implementation methods acceptable or if their satisfaction influences CR outcomes. We used mixed participatory methods, including focus groups, to co-develop a CR satisfaction scale. This was refined using three psychometric criteria (Cronbach’s alpha, item discrimination, test-retest agreement) to select items. Factor analysis explored potential substructures. The refined measure was used in structural equation joint modelling to evaluate whether satisfaction with CR is affected by implementation method and treatment engagement or influences recovery outcome, using data from a randomised controlled trial. Four themes (therapy hours, therapist, treatment effects, computer use) generated a 31-item Cognitive Remediation Satisfaction scale (CRS) that reduced to 18 Likert items, 2 binary and 2 open-ended questions following psychometric assessment. CRS had good internal consistency (Alpha = 0.814), test-retest reliability (*r*= 0.763), and concurrent validity using the Working Alliance Inventory (*r* = 0.56). A 2-factor solution divided items into therapy engagement and therapy effects. Satisfaction was not related to implementation method but was significantly associated with CR engagement. Therapy hours were significantly associated with recovery, but there was no direct effect of satisfaction on outcome. Although satisfaction is important to therapy engagement, it has no direct effect on outcome. CR therapy hours directly affect outcome irrespective of which implementation model is used, so measuring satisfaction early might help to identify those who are likely to disengage. The study has mixed methods design.

## Introduction

There is robust evidence that Cognitive Remediation (CR) interventions are effective (e.g.^1^), and that four key ingredients^2^ can boost cognitive and functional outcomes^3–5^. From the users’ point of view, CR is associated with perceived cognitive improvement^6–9^ as well as increased confidence and motivation^7–9^. We know little about how to implement this therapy successfully as meta-analyses have not found differences between implementation methods (e.g., Independent, Group and One-to-One). One variable that might tip this balance is the view of the CR users, and particularly their satisfaction with treatment. Satisfaction may affect treatment engagement which has an influence on the critical dose of therapy received and therefore the treatment outcome, and so may even suggest the most efficient way of providing treatment for most service users. Alternatively, the outcome of treatment may affect satisfaction.

Acceptability and satisfaction with treatment are usually assessed indirectly by treatment drop-out and hours of engagement, rarely through direct satisfaction measures^5,10,11^. Even when a satisfaction measure is used, it usually answers questions posed by clinical researchers and not those generated by the potential therapy users. Our approach was to co-develop a Patient Reported Outcome Measures (PROM) using iterative, participatory methods^12,13^ focussing on questions important to the participants themselves. This psychometrically sound measure of CR treatment satisfaction (Cognitive Remediation Satisfaction (CRS) scale) was then used to investigate the potential effects of satisfaction on treatment engagement, implementation and the primary treatment outcome, personal recovery goals, from a completed randomised clinical trial (RCT), ECLIPSE^14^ which showed improvements in recovery (measured by the Goal Attainment Scale^15^, following CR with CIRCuiTS^TM^ software^16^.

## Method

### Design

This is a mixed methods study involving two stages: 1) Development of the psychometrically sound Cognitive Remediation Satisfaction scale (CRS); and 2) applying the CRS to evaluate satisfaction with cognitive remediation (CR) implementation methods and outcomes in a secondary analysis of data from an RCT involving three different CR implementation techniques (one-to-one, group and independent use^14,16^). These data showed overall functional improvement post-treatment for the one-to-one and group methods but not the independent method. Ethical approval for measure development was obtained from Chelsea Research Ethics Committee (REC; 15/LO/1816) and for the RCT from Camden and Kings Cross REC (15/LO/ 1960).

### Participants and procedures

#### Stage 1: CRS development

Participants aged 18-35, and able to provide informed consent were recruited from early intervention services (EIS) to two focus groups facilitated by service user researchers. After familiarising participants with CR, they discussed the content and format of a new satisfaction scale. The topic guide was based on our existing co-developed CR measure designed for paper-and-pencil CR^9,17^. The focus groups considered the items from the original scale and adapted and added further items. An initial thematic analysis of the transcribed focus group interviews generated a draft questionnaire that was discussed in the second focus group for respondent validation and analysed thematically using NVIVO12 to produce a revised version. The draft CRS was completed online at the end of therapy (post-treatment) in a randomised controlled trial and participants were offered the opportunity to repeat it with a one-to-two-week interval. The draft CRS was then subjected to psychometric testing to refine the ordinal items and produce the final version (see analysis).

#### Stage 2: Assessing post-CR satisfaction

**Fig. 1:**
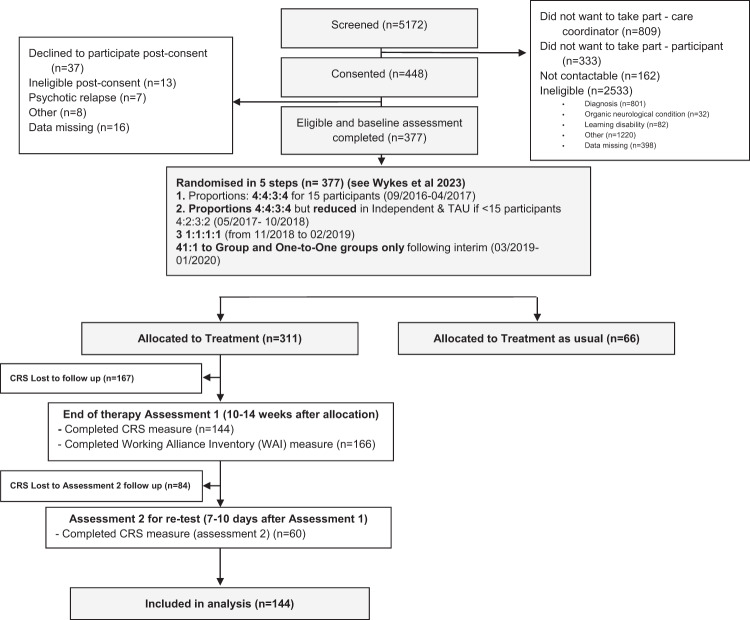
Completion of the draft CRS within the RCT.

Figure 1 shows the completion of the draft CRS within the RCT. Participants were aged 16 to 45, had attended UK NHS EIS for at least three months, had a diagnosis of non-affective psychosis and could provide informed consent. Exclusion criteria were an inability to communicate in English, an underlying organic/neurological condition, and a co-morbid diagnosis of learning disability. Participants were randomised to receive the same number of therapy sessions with the CR computer software, CIRCuiTS^TM^, but with variation in the amount of therapist support available to an individual participant^14,18^.

##### Assessments

We used the baseline measures from the RCT data to assess the robustness of the CRS data collected in case those that completed the measure were different from the remaining sample by considering: demographic variables, total negative symptoms (CAINS^19^), and global psychopathology (PANSS total^20^), and a global cognition composite score derived from a range of measures^14^.

To examine convergent validity and the exploration of satisfaction associations, we collected the Working Alliance Inventory (WAI^21^) online. The WAI is a 36-item measure of therapy engagement that assesses different aspects of therapy satisfaction. In addition, we used the total number of CR hours in valid sessions as a measure of treatment engagement and the primary outcome (post-treatment Goal Attainment Scale (GAS) weighted T-score^15^ that measured improvements in personally defined recovery goals.

In addition, we asked if participants thought they had improved their concentration, memory, being alert, confidence, problem solving, planning, social interaction, setting goals and motivation. All were rated on a scale of 0 = not at all to 4 = A lot and we added the scores to get a total subjective improvement score. This allowed us to consider if different implementation methods produced more subjective improvement.

### Implementation methods

The CR adopted is a therapist-supported computerised therapy that embeds metacognitive aspects of learning into the software. CIRCuiTS^TM^ is feasible and acceptable and has had independent evaluations^17,18,22,23^ Although all groups had access to CR^17^ for up to 42 sessions, they differed in their access to a therapist. In the *independent* method, the participant had access to an introductory session and then half an hour a week of therapist contact. For *group* and *one-to-one* methods, the therapist was always in contact, but in the group method the therapist’s time was shared with up to four other people.

### Lived experience involvement

Lived experience involvement is associated with study success^24^ and so we consulted people with experience of using mental health services at every stage. The development of the RCT and the study protocol involved service users and we also continued their involvement as advisors (Patient Advisory Board), critical reviewers, and authors of this publication in addition to employing researchers with a background in using mental health services.

### Analysis

#### Qualitative analysis

Data were transcribed after each focus group and analysed independently by two service user researchers, using inductive thematic analysis^25^ on NVIVO12. This involved familiarisation with the data, generation of codes and then development, refinement, and definition of sub- and over-arching themes. Any differences were resolved through discussion.

We also analysed the themes arising for each implementation method in the same way from answers to any open-ended questions included in the final measure.

#### Data used and sensitivity analyses

Draft CRS and WAI scores were pro-rated if fewer than 20% item scores were missing. Since the CRS was completed by only a subset of trial participants, logistic regression was used to examine whether missing draft CRS scores were related to CR method, trial site, demographic characteristics, cognition, and symptoms. Any variables that were predictive of missingness were included in subsequent analyses.

#### Scale refinement and validity

For Likert scale items, three psychometric criteria for item selection from the draft CRS were used. CRS: item-rest correlation (Cronbach’s alpha), item discrimination parameter from a graded membership item-response theory model, and test-retest (kappa; intra-class correlation). The test information curve provided an understanding of measure information across the range of scores. After refining the scale, we explored potential sub-structures with a factor analysis using the item polychoric correlation matrix. Validity was assessed on the final scale through Pearson correlations between the post-treatment refined CRS score and: (i) treatment engagement (therapy hours completed), where it was assumed that those more satisfied would be more likely to take part in more therapy hours during the treatment window, and (ii) the WAI score measures aspects of treatment satisfaction but, before analysis, we removed the four CRS therapist items that directly overlapped with the WAI. Higher total CRS scores reflect better satisfaction so, where necessary, some items were reverse scored. The absence of cultural bias was tested by gender and ethnicity.

We also assessed whether the CRS score provided more information than a single question “Overall were you satisfied with treatment” as a high correlation suggests that a simplified measure would be just as appropriate.

Following refinement, we calculated the mean CRS score for each implementation method.

#### The relationship between satisfaction, implementation models and trial outcome

We first used an ANOVA model to investigate whether satisfaction scored using the post-treatment refined CRS was related to implementation method. The model included trial arm, site, and variables identified as predictive of missingness.

Analyses of potential models of the relationships between satisfaction, implementation method and outcome used structural equation joint modelling (Stata 17 sem, method mlmv; vce(robust); StataCorp 2019) to account for selective drop-out by using all CR recipients to examine the effect of satisfaction on GAS at 15-weeks post-therapy. In these models we assessed whether any effect of satisfaction on post-therapy outcome was due to either the implementation method or to higher levels of treatment engagement (hours of CR). The models were refitted, using each of the newly defined satisfaction component factors to determine whether either component affected the outcome. Sensitivity analyses included: (i) refitting the model with log transformed treatment engagement as measured using hours of CR to address non-normality of the distribution and (ii) investigating potential effects at a 6-month follow-up rather than post-treatment (known as sleeper effects) by replacing GAS at 15-weeks with GAS at 6-months.

## Results

### Stage 1: CRS development

#### Sample characteristics

The same 8 participants took part in the two focus groups. All had a diagnosis of non-affective psychosis and were from a minority background; seven were men and the median age was 28.

#### Draft CRS content

For item format, focus group participants mentioned difficulties they had with attention, concentration, memory, and planning (the cognitive targets of CR) and said that they preferred closed questions with set response options but with added comment boxes for fuller answers for some questions. The derived draft CRS included 24 items on a Likert scale, 5 binary-categorical and two open-ended questions with optional free text space.

There were four key item themes: *therapy hours* (understanding and use of different CR components); *the therapist* (understanding the therapist’s role and levels of support); the *therapy effects* (applying skills learnt from CR into everyday life and the personal impact of undertaking and ending the therapy); and *using the computer* (ease of computer use and the CR programme). The 2 open-ended items asked about the most helpful and least helpful aspects of CR. Examples from the thematic analysis, used to develop item content, are provided in the supplement. During the respondent validation process, there was consensus that the draft scale was comprehensive and of an appropriate length and wording.

#### Data and participants

Table 1 shows demographic and baseline characteristics for the 144 trial participants who received CR via the CIRCuiTS^TM^ programme and completed the draft satisfaction scale at post-treatment. Logistic regression showed that the response rate was only associated with living with a partner and living with a parent. Both variables became non-significant once account was taken of the post-baseline measure of completed CR hours, and therefore were not included in the structural equation models.

**Table 1:** Demographic and baseline characteristics of participants completing the draft CRS.

Implementation Method				
	Independent (*n* = 23)	Group (*n* = 58)	One-to-One (*n* = 63)	All participants (*n* = 144)
Age at consent Mean (SD)	26.64 (4.93)	26.28 (6.25)	26.57 (6.24)	26.47 (6.02)
Sex N (%)				
Male	15 (65.22%)	41 (70.69%)	52 (82.54%)	108 (75.00%%)
Female	8 (34.78%)	17 (29.31%)	11 (17.46%)	36 (25.00%)
Ethnicity N (%)				
White	13 (56.52%)	23 (39.66%)	29 (46.03%)	65 (45.14%)
Black (African, Caribbean)	7 (30.43%)	21 (36.21%)	16 (25.40%)	44 (30.56%)
Asian (Bangladeshi, Indian, Pakistani)	1 (4.35%)	8 (13.79%)	10 (15.87%)	19 (13.19%)
Other	2 (8.70%)	6 (10.34%)	7 (11.11%)	15 (10.42%)
* Missing*	0 (0.00%)	0 (0.00%)	1 (1.59%)	1 (0.69%)
Employment status N (%)				
Unemployed	17 (73.9%)	36 (62.1%)	44 (69.8%)	97 (67.4%)
Active	6 (26.1%)	22 (37.9%)	19 (30.2%)	47 (32.6%)
Living situation N (%)				
Own property (private, rented)	8 (34.7%)	13 (22.4%)	19 (30.2%)	40 (27.8%)
With parents	15 (65.2%)	45 (77.6%)	44 (69.8%)	104 (72.2%)
Relationship status N (%)				
Single / Divorced	19 (82.6%)	52 (89.7%)	54 (85.7%)	125 (86.8%)
In relationship	4 (17.4%)	6 (10.3%)	9 (14.3%)	19 (13.2%)
Site				
01	3 (13.0%)	8 (13.8%)	10 (15.9%)	21 (14.6%)
02	4 (17.4%)	7 (12.1%)	10 (15.9%)	21 (14.6%)
03	2 (8.7%)	3 (5.2%)	2 (3.2%)	7 (4.9%)
04	3 (13.0%)	4 (6.9%)	5 (7.9%)	12 (8.3%)
05	4 (17.4%)	21 (36.2%)	20 (31.8%)	45 (31.3%)
06	7 (30.4%)	15 (25.9%)	16 (25.4%)	38 (26.4%)
GAS T-score Mean (SD)	33.36 (4.47)	32.92 (4.64)	32.65 (4.81)	32.87 (4.66)
Composite cognitive score Mean (SD)	-1.06 (5.04)	-0.67 (5.32)	1.54 (5.09)	0.24 (5.27)
PANSS Total score Mean (SD)	60.59 (23.14)	56.93 (13.75)	56.29 (16.02)	57.22 (16.41)
CAINS Total score Mean (SD)	20.35 (10.46)	18.55 (10.43)	18.37 (9.53)	18.76 (10.00)
Post baseline measures at trial endpoint				
Completed CR N (%)	23 (100.0%)	58 (100.0%)	63 (100.0%)	144 (100.0%)
Hours of CR Mean (SD)	14.82 (8.04)	24.14 (9.35)	26.39 (9.64)	23.64 (10.17)
*CRS Mean (SD)	85.84 (9.34)	88.04 (8.32)	87.56 (9.65)	87.48 (9.05)
GAS T-score Mean (SD)	47.42 (9.24)	53.72 (11.90)	51.13 (10.98)	51.66 (11.26)
Working Alliance Inventory Mean (SD)	213.30 (23.12)	217.95 (24.15)	212.66 (28.48)	214.88 (25.96)
Satisfied overall N (%)	22 (95.65%)	57 (98.28%)	59 (93.65%)	138 (95.83%)
Valued therapist support N (%)	23 (100.0%)	58 (100.0%)	62 (98.41%)	143 (99.31%)

*Refined CRS score.

#### Psychometric analysis and scale refinement

Both item-rest correlations from a Cronbach analysis and item discrimination estimates from an ordinal IRT model showed that four of the 24 Likert-scale items in the draft CRS performed markedly poorer and were removed (See Table 2) and one further item (Q2) did not provide good test-retest reliability. For the **total score** we also removed another item - the Overall Satisfaction Score - leaving 18 well-performing Likert Scale items (mean=87.48, SD = 9.05, median=87.94, range= 56-108; Cronbach alpha 0.814; test-retest intraclass correlation 0.763). An IRT test information curve (Figure S1, Supplement) indicates that items provided more precise estimates of satisfaction over the lower two-thirds of the score distribution suggesting that the scale is better at measuring lower than higher satisfaction.

**Table 2:** Eligible Likert satisfaction items in the draft CRS prior to refinement and excluding the overall satisfaction item.

Items in bold included in final CRS Score					
Shortened item description	N (Test-retest n)	Item-rest	Item discrimination	Test-retest kappa	Kappa p-value
I felt confident with the CR programme	145 (59)	0.1943	0.565959	0.472	<0.001
Understanding strategy use	145 (60)	0.4468	1.624891	0.119	0.189
Using strategies in real life	147 (60)	0.2963	0.902416	0.619	<0.001
Difficulty of some tasks or exercises	147 (60)	0.0413	-0.22451	0.650	<0.001
Tasks and exercises were rated too often	147 (60)	0.0969	-0.28305	0.667	<0.001
I was sorry when therapy ended	145 (59)	0.3959	0.953287	0.775	<0.001
The computer/tablet were easy to use	145 (59)	0.269	0.919951	0.801	<0.001
I learnt how to use a computer/tablet	146 (59)	-0.0875	-0.12951	0.798	<0.001
The CR programme was easy to use	147 (60)	0.4802	1.699704	0.524	<0.001
I needed extra computer support	147 (60)	-0.0389	-0.39503	0.655	<0.001
I understood the therapist role	146 (60)	0.3619	1.045312	0.501	<0.001
I got on well with my therapist	146 (60)	0.3847	2.608366	0.614	<0.001
My therapist was a good teacher	146 (60)	0.4905	3.097226	0.584	<0.001
The therapist and I could feedback to each other	145 (59)	0.5023	3.078856	0.461	<0.001
I valued the therapist support	146 (60)	0.5043	3.964249	0.349	0.003
Therapy occupied my mind	144 (60)	0.454	1.184183	0.611	<0.001
Therapy occupied me	144 (60)	0.2393	0.648953	0.560	<0.001
I enjoyed CR therapy	143 (60)	0.4911	1.33705	0.524	<0.001
CR skills have helped me	143 (60)	0.4536	1.242615	0.484	<0.001
Therapy made me aware of weaknesses	136 (57)	0.36	1.077743	0.462	<0.001
Therapy made me feel better	142 (59)	0.6098	1.89365	0.484	<0.001
CR helped me improve my everyday life	142 (59)	0.4938	1.186436	0.642	<0.001
I would change the CR therapy	140 (58)	0.3936	-1.08923	0.689	<0.001

Categorical factor analysis gave eigen-values of 6.04, 2.37 and 1.10 for the first 3 factors. To find the smallest number of interpretable factors that explain the maximum amount of variability in the data, a two-factor solution was chosen which explained 73% of the variability in the 18 items. Table S2 and Figure S2 give factor loadings for the 2-factor solution, suggesting the items divide into therapy engagement and therapy effects. The 18-item total score was correlated with treatment hours (*r* = 0.22, *p* = 0.007) completed during the therapy window (*r* = 0.187, *p* *=* 0.025 with log-treatment hours). The correlation with WAI score (*r* = 0.56, *p* < 0.001) suggested significant and reasonable convergent validity. CRS total was unrelated to study site (5df *p* = 0.340), baseline negative symptoms (*p* = 0.274), baseline PANSS (*p* = 0.740) and the cognitive composite (*p* = 0.845). The 18-item total score was correlated, but weakly, with the “Overall satisfaction” item of 0.31 (polyserial, *p* = 0.001) suggesting that the total score does provide an adequate picture of people’s experiences. Analysis by demography showed a tendency for women to be more satisfied than men (*p* = 0.021) and some variation by ethnicity (3df chi^2^ *p* = 0.062) with higher mean scores among Asian participants (91.42) than White (87.43), Black (86.57), or other ethnicities (84.88).

There were two binary items included in the CRS. The first asked whether CR had been helpful and the other asked whether another implementation method would have been preferable. Not all participants completed this section of the CRS but of those that did a large number (95.7%, *N* = 111) thought that CR was helpful and only 6 (4.6%, *N* = 129) would have preferred another method with one person indicating their preference (from group to one-to-one). There were few differences between the implementation methods, although one-to-one therapy was found to be more helpful and preferred (One-to-One 98.6%, 97.5%; Group 92%, 90.9%; Independent 87.55, 94% respectively).

The two open-ended questions followed the binary items and asked participants to expand on their answers to say why they did or did not think CR was helpful and why they preferred or not their method of delivery. The themes from the answers were analysed separately for each implementation method. Participants receiving CR on an independent basis appreciated the flexibility and choice it provided (“*It gave me freedom to choose when I did the therapy”; “I was able to focus alone”)*. However, some found it difficult because of lack of motivation “*I wasn’t able to set myself targets to get the tasks done on time”*.

Group participants appreciated the opportunity to share experiences and views with their peers, although sometimes there was a lack of group engagement that reduced interaction and attending could be anxiety-provoking. They felt that group delivery had a positive impact on motivation. For instance, one person reported that *“We could bounce ideas off each other and we got more input from different people than you would have got in a one-to-one setting”* but another said, “*In the group we didn’t talk to each other, we got on with our own thing”*.

Participants who received CR on a one-to-one basis reported a strong therapeutic relationship and appreciated the support provided, particularly at the outset of therapy (“*I needed someone holding my hand at first, especially since the tasks seemed too simple to warrant my attention in the beginning”*). Having therapy on this basis helped with focus and level of understanding, as well as reducing anxiety and increasing confidence levels (e.g., *Having someone to confide in and to go out and about with helped me with my anxiety)*.

#### Is satisfaction related to implementation type or treatment outcome

There was no significant effect of implementation method on satisfaction (means Independent 85.84, Group 88.04, One-to-One 87.56; 2df F-test *p* = 0.606). Subjective improvement from therapy was significantly correlated with the CRS score (0.55, *p* < 0.01), but when investigated by each implementation method only the correlations with Group (0.643 *p* < 0.01) and One-to-One (0.55 *p* < 0.01) were significant, but the Independent method correlation was low (0.102, n.s.).

**Fig. 2:**
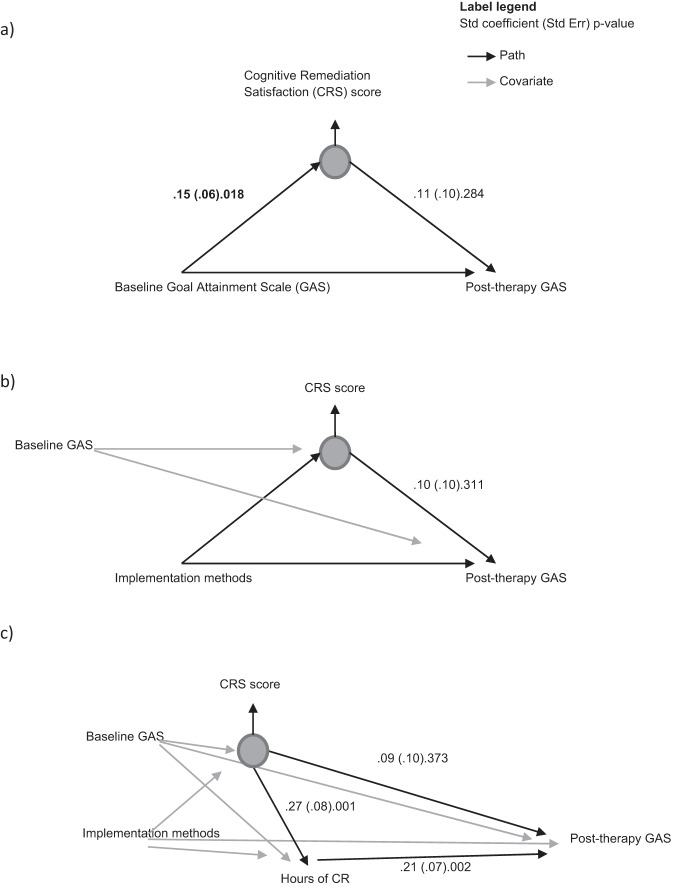
A series of models of the relationship of satisfaction to GAS outcome. **a** The simple model of a direct association. **b** The additional effect of implementation methods. **c** The additional effect of engagement (number of hours of therapy).

Figure 2 and Table S1 shows a series of models and estimated effects examining the role of satisfaction on treatment outcome. The simple model (Fig. 2a) indicates no significant association between satisfaction and GAS improvement. Model 2b shows no significant association in satisfaction between the implementation methods (Independent: Group, *p* = 0.312; One-to-One: Group, *p* = 0.847; Independent: One-to-One, *p* = 0.402), and a non-significant association between satisfaction and GAS improvement (*p* = 0.311). In Model 2c therapy engagement (therapy hours completed) was added and found to be significantly associated with satisfaction (*p* = 0.001) and with GAS improvement (*p* = 0.002), but the direct effect of satisfaction on GAS changed little and remained non-significant (*p* = 0.373).

When the satisfaction component factor scores were used rather than the overall score, neither of the two additional analyses found a significant association between satisfaction and improvement in the GAS outcome (Tables S3 and S4).

None of the sensitivity analyses found any significant relationship between satisfaction and improvement in outcome (*p* = 0.346) after (i) log transforming therapy engagement (Table S5) or (ii) the GAS outcome measured at 6 months (*p* = 0.964, Table S6), and at that point the relationship between outcome and therapy engagement was also marginal (*p* = 0.058).

## Discussion

We co-developed and refined a psychometrically robust CR satisfaction measure driven by service user views. The measure was also superior to asking a single question on overall satisfaction. One of the key themes to arise from the qualitative work was the important role of the therapist, which is reflected in four CRS items. The professional and interpersonal skills of the therapist and a strong working alliance are consistently cited as important influences on therapy outcomes in existing literature^7,9,26^. This is also supported in this study as almost all participants were satisfied with their therapist (95.8%), valued their support (99.3%), and felt they had a good working alliance (Table 2).

Service users in the focus groups prioritised the functional, cognitive, and psychological effects of CR, reaffirming existing literature^26,27^. Prior research has shown general acceptability across different computerised therapies, although lack of access and skills can impede progress, highlighted also by our focus group participants^8,17^. The final CRS asks about difficulties using computers or tablets, the computerised CR programme and whether any support is needed. Although the items relating only to computer use were removed from the total score, they are retained in the scale as this was important to service users and may shed light on acceptability as some software may be more complex or easier than the one tested here. A key issue for focus group members is ensuring that the CRS could be completed by people who might be experiencing cognitive difficulties associated with psychosis.

We tested the draft CRS on those who entered a trial and the individuals who completed the measure were representative of those attending UK Early Intervention Services so we expect that these results will generalise to people using those services. We also did not find any significant difference between those who completed the measure and those who received therapy but did not complete the measure.

We did expect that there would be a correlation between the overall subjective improvement from CR and satisfaction with treatment and this was true for the Group and One-to-One methods but not for Independent therapy. It may be that the presence of a therapist allows an individual to develop better awareness of their improvements by supporting metacognitive knowledge via interactions. The results of the open-ended questions suggests that discussions with the therapist were key considerations for both group and one-to-one participants. Of interest is that the engagement with the independent therapy was much lower compared to the other conditions and the participants did report how hard it was to motivate themselves and set goals when receiving CR by this method.

### Relationship of Satisfaction to CR implementation and outcomes

Satisfaction, as expected, was related to therapy engagement as measured by hours completed but was not associated with how you completed those therapy hours – independently, in a group or one-to one. However, those receiving CR by the independent method completed on average far fewer therapy hours (14.8 vs 24.0 or 26.0), so despite satisfaction not being related to implementation, there was a relationship of implementation and therapy engagement. In the independent method access to a therapist was limited to an hour every two weeks and this may have affected engagement. Therapist effects on engagement have been emphasised by others^28,29^. However, although a recent meta-analysis demonstrated that active therapists affect CR benefit significantly^5^, the same group found, in a further meta-analysis, that there were no independent effects of an active therapist on treatment drop-out^10^.

While satisfaction was significantly associated with therapy hours, and hours were significantly associated with GAS improvement, the direct effect of satisfaction on GAS improvement was not significant. This is surprising as we would assume that the effects of therapy on outcome would be related to satisfaction with treatment but the association between outcome and satisfaction could be wholly explained through increasing engagement although other possible causal routes are possible. Practice with CR together with progressive achievement of outcome goals and improved performance on the CR tasks might all increase satisfaction as well as increasing confidence and self-esteem. The causal ordering among satisfaction, engagement and goal attainment also cannot be firmly established because they were measured at the same time, but the results suggest that satisfaction may not be central to the causal mechanism of increasing functioning but may better be considered a secondary desirable outcome, relating to treatment engagement.

#### Strengths and Limitations

This is the largest randomised study of computerised CR in EIS that also included a co-developed satisfaction measure. We used both classical and modern psychometric methods and chose our exploratory analyses methods to understand whether completion rates could affect our data. A larger study would increase our confidence in the factor analysis results. We could not however, implement an instrumental variable approach that would have allowed for post-randomisation confounding which would have permitted a stronger attribution of causality. We only tested implementation method and did not contrast different CR software which is important for services choosing specific therapies especially if it incurs a charge. A comparison study would be the next research step.

## Conclusions

Evidence based treatments can only be successfully implemented if they are acceptable to service users^30^ and satisfaction is a key dimension of acceptability. The CRS development highlighted four elements in two factors - therapy engagement and therapy effects and the quantitative analyses show that satisfaction is related to therapy engagement but not to recovery. Satisfaction is related to engagement which is related to benefits and although we have not detected a causal relationship, measuring satisfaction is still important for engaging service users in health services. It is also a way to detect unwanted effects and potentially to improve service efficiency and effectiveness if this measure can begin to detect those at risk of disengagement. We have produced a measure to detect those potential unwanted effects.

### Supplementary information

Supplement
