# Supplementary material for: Satisfaction with cognitive remediation therapy: its effects on implementation and outcomes using the cognitive remediation satisfaction scale
Source: Schizophrenia (Heidelb). 2023 Sep 30;9(1):67. doi: 10.1038/s41537-023-00390-9 (PMC10542804; doi:10.1038/s41537-023-00390-9)
Supplement: Supplementary file 1 — Supplement [file 41537_2023_390_MOESM1_ESM.docx]

**SUPPLEMENT**

***Examples of thematic analysis to support item content***

Service users felt that it was important to capture levels of initial confidence in being able to use the software as it may affect their motivation and ability to undertake the therapy, as well as being a potential indicator of the level of support they were receiving.

*“You’d be like ‘no, this is too much for me’. Some of it I’d be trying to do but I think to myself ‘you’ve got to be like a rocket genius to actually get it all right’”. (SU2)*

A new item was therefore added: ‘I felt confident when I first saw the CR programme’.

Support from the therapist was seen as vital, as participants said they were likely to miss out tasks or difficult exercises if left alone. Clear explanations and support would address their lack of confidence, which led to the item: ‘I valued the support my therapist gave me during CR’.

Although it was assumed that young people were comfortable using technology, some participants felt this was not always true, particularly for people who might be experiencing cognitive difficulties. This led to the inclusion of ‘I needed extra computer support during the therapy sessions’.

*“A lot of people in our age group, they usually go online or use computers anyway or IPADs.” (SU1)*

*“I think we kind of take it for granted but there’s [lots] of people that aren’t”. (SU7)*

Participants found certain questions sensitive, especially about their relationship with the therapist, and were reluctant to answer them.

*“Oh yeah, ‘did you miss him or her when the therapy ended’. It just felt like it’s a bit personal, like you don’t know how to, what to say.” (SU4)*

They still thought it was important to ask about the therapeutic relationship and so included the item, ‘I got on well with my therapist’.

Some suggested items from the previous measure provoked a discussion about terminology and the meaning of words like disability and limitations. There was agreement that the word ‘limitations’ was problematic, and they preferred ‘weaknesses’. Limitations were thought to be negative choices or impossible actions that could not be controlled, whereas ‘weaknesses’ could be identified and worked on in a therapeutic setting, so the item was reworded.

*“I think, I think it’s better this way because your weaknesses are things that someone can help you overcome” (SU4)*

| Table S1: SEM standardized structural coefficient estimates three models in Figure 2A, 2B, and 2C | | |
| --- | --- | --- |
|  | **Standardized Estimate (95% CI)** | **p-value** |
| Model 1 (Figure 2A) | | |
| Post-therapy GAS t-score: |  |  |
| CRS score | 0.11 (-0.088, 0.300) | 0.284 |
| Baseline GAS t-score | **0.15 (0.025, 0.265)** | **0.018** |
| CRS score: |  |  |
| Baseline GAS t-score | 0.06 (-0.095, 0.217) | 0.444 |
| Model 2 (Figure 2B) | | |
| Post-therapy GAS t-score: |  |  |
| CRS score | 0.10 (-0.092, 0.290) | 0.311 |
| Independent CRT | **-0.20 (-0.333, -0.058)** | **0.005** |
| One-to-One CRT | -0.03 (-0.177, 0.108) | 0.637 |
| Baseline GAS t-score | **0.16 (0.039, 0.276)** | **0.009** |
| CRS score: |  |  |
| Independent CRT | -0.10 (-0.289, 0.092) | 0.312 |
| One-toOne CRT | -0.02 (-0.183, 0.150) | 0.847 |
| Baseline GAS t-score | 0.07 (-0.089, 0.220) | 0.406 |
| Model 3 (Figure 2C) | | |
| Post-therapy GAS t-score: |  |  |
| CRS score | 0.09 (-0.105, 0.278) | 0.373 |
| Hours of CR | **0.21 (0.074, 0.339)** | **0.002** |
| Baseline GAS t-score | **0.17 (0.055, 0.277)** | **0.003** |
| Independent CRT | **-0.15 (-0.289, -0.015)** | **0.029** |
| One-to-One CRT | -0.07 (-0.214, 0.070) | 0.323 |
| CRS score: |  |  |
| Independent CRT | -0.06 (-0.261, 0.133) | 0.524 |
| One-to-One CRT | 0.01 (-0.155, 0.174) | 0.912 |
| Baseline GAS t-score | 0.03 (-0.120, 0.187) | 0.669 |
| Hours of CR: |  |  |
| CRS score | **0.27 (0.111, 0.427)** | **0.001** |
| Independent CRT | **-0.16 (-0.275, -0.045)** | **0.006** |
| One-to-One CRT | **0.18 (0.063, 0.295)** | **0.003** |
| Baseline GAS t-score | -0.05 (-0.156, 0.058) | 0.366 |

**Figure S1 Test Information Curve**

**Figure S2 Factor Loading scatter diagram.**

| **Table S2 Rotated Factor Loadings** | | | |
| --- | --- | --- | --- |
| **Item** | **Shortened item description** | **Factor1** | **Factor2** |
| q1 | felt confident with the CR programme | 0.3413 | - |
| q3 | Using strategies in real life | - | 0.3686 |
| q8 | I was sorry when therapy ended | - | 0.4087 |
| q9 | The computer/tablet were easy to use | 0.4132 | - |
| q11 | The CR programme was easy to use | 0.5761 | - |
| q13 | I understood the therapist role | 0.5494 | - |
| q14 | I got on well with my therapist | 0.9765 | - |
| q15 | My therapist was a good teacher | 0.8233 | - |
| q16 | The therapist and I could feedback to each other | 0.9005 | - |
| q17 | I valued the therapist support | 0.7837 | - |
| q18 | Therapy occupied my mind | - | 0.8359 |
| q19 | Therapy occupied me | - | 0.8177 |
| q20 | I enjoyed CR therapy | - | 0.687 |
| q21 | CR skills have helped me | - | 0.7368 |
| q22 | Therapy made me aware of weaknesses | - | 0.5391 |
| q23 | Therapy made me feel better | 0.299 | **0.5624** |
| q26 | CR helped me improve my everyday life | - | 0.7508 |
| q27 | I would change the CR therapy | - | 0.4411 |

| Table S3: Factor 1 SEM standardized structural coefficient estimates for the three models | | |
| --- | --- | --- |
|  | **Standardized Estimate (95% CI)** | **p-value** |
| Model 1 | | |
| Post-therapy GAS t-score: |  |  |
| CRS score | 1.52 (-0.40, 3.44) | 0.121 |
| Baseline GAS t-score | **0.36 (0.07, 0.65)** | **0.016** |
| CRS score: |  |  |
| Baseline GAS t-score | 0.07 (-0.05, 0.20) | 0.259 |
| Model 2 | | |
| Post-therapy GAS t-score: |  |  |
| CRS score | 0.12 (-0.05, 0.28) | 0.175 |
| Independent CRT | **-0.19 (-0.33, -0.05)** | **0.008** |
| One-to-One CRT | -0.03 (-0.17, 0.12) | 0.713 |
| Baseline GAS t-score | **0.15 (0.03, 0.27)** | **0.013** |
| CRS score: |  |  |
| Independent CRT | -0.14 (-0.32, 0.05) | 0.160 |
| One-to-One CRT | -0.07 (-0.23, 0.09) | 0.367 |
| Baseline GAS t-score | 0.10 (-0.06, 0.25) | 0.226 |
| Model 3 | | |
| Post-therapy GAS t-score: |  |  |
| CRS score | 1.32 (-0.57, 3.22) | 0.171 |
| Hours of CR | **0.20 (0.09, 0.31)** | **<0.001** |
| Baseline GAS t-score | **0.38 (0.09, 0.67)** | **0.009** |
| Independent CRT | **-5.36 (-9.19, -1.54)** | **0.006** |
| One-to-One CRT | -0.78 (-4.14, 2.58) | 0.649 |
| CRS score: |  |  |
| Independent CRT | -0.33 (-0.82, 0.15) | 0.181 |
| One-to-One CRT | -0.15 (-0.49, 0.19) | 0.384 |
| Baseline GAS t-score | 0.019 (-0.01, 0.05) | 0.259 |
| Hours of CR : |  |  |
| CRS score | **3.69 (3.23, 4.16)** | **<0.001** |
| Independent CRT | **-1.23 (-3.03, 0.58)** | **0.183** |
| One-to-One CRT | **-0.56 (-1.83, 0.71)** | **0.391** |
| Baseline GAS t-score | 0.07 (-0.05, 0.20) | 0.258 |

| Table S4: Factor 2 SEM standardized structural coefficient estimates for the three models | | |
| --- | --- | --- |
|  | **Standardized Estimate (95% CI)** | **p-value** |
| Model 1 | | |
| Post-therapy GAS t-score: |  |  |
| CRS score | 0.72 (-1.64, 3.08) | 0.548 |
| Baseline GAS t-score | **0.36 (0.07, 0.66)** | **0.015** |
| CRS score: |  |  |
| Baseline GAS t-score | 0.07 (-0.16, 0.30) | 0.569 |
| Model 2 | | |
| Post-therapy GAS t-score: |  |  |
| CRS score | 0.71 (-1.61, 3.03) | 0.548 |
| Independent CRT | **-5.69 (-9.52, -1.86)** | **0.004** |
| One-to-One CRT | -0.82 (-4.18, 2.55) | 0.635 |
| Baseline GAS t-score | **0.39 (0.10, 0.69)** | **0.008** |
| CRS score: |  |  |
| Independent CRT | -1.12 (-4.23, 2.00) | 0.482 |
| One-to-One CRT | -0.004 (-2.47, 2.46) | 0.997 |
| Baseline GAS t-score | 0.07 (-0.16, 0.30) | 0.537 |
| Model 3 | | |
| Post-therapy GAS t-score: |  |  |
| CRS score | 1.38 (-0.86, 3.62) | 0.228 |
| Hours of CR | **0.19 (0.06, 0.32)** | **0.004** |
| Baseline GAS t-score | 0.39 (0.10, 0.67) | 0.009 |
| Independent CRT | -5.37 (-9.18, -1.55) | 0.006 |
| One-to-One CRT | -0.75 (-4.10, 2.59) | 0.658 |
| CRS score: |  |  |
| Independent CRT | -0.43 (-3.65, 2.79) | 0.794 |
| One-to-One CRT | 0.44 (-2.00, 2.87) | 0.725 |
| Baseline GAS t-score | 0.02 (-0.21, 0.24) | 0.880 |
| Hours of CR : |  |  |
| CRS score | **4.23 (2.16, 6.30)** | **<0.001** |
| Independent CRT | **-5.56 (-8.81, -2.32)** | **0.001** |
| One-to-One CRT | **4.83 (1.61, 8.04)** | **0.003** |
| Baseline GAS t-score | -0.11 (-0.41, 0.19) | 0.480 |

| Table S5: SEM standardized structural coefficient estimates for the three models with Hours of CR log transformed | | |
| --- | --- | --- |
|  | **Standardized Estimate (95% CI)** | **p-value** |
| Model 3 | | |
| Post-therapy GAS t-score: |  |  |
| CRS score | 0.095 (-0.10, 0.29) | 0.346 |
| Log(Hours of CR) | **0.220 (0.07, 0.38)** | **0.005** |
| Baseline GAS t-score | **0.159 (0.05, 0.27)** | **0.005** |
| Independent CRT | **-0.162 (-0.30, -0.03)** | **0.019** |
| One-to-One CRT | -0.070 (-0.21, 0.07) | 0.326 |
| CRS score: |  |  |
| Independent CRT | -0.074 (-0.26, 0.12) | 0.445 |
| One-to-One CRT | 0.033 (-0.13, 0.20) | 0.689 |
| Baseline GAS t-score | 0.036 (-0.12, 0.19) | 0.645 |
| Log (Hours of CR): |  |  |
| CRS score | **0.372 (0.09, 0.65)** | **0.009** |
| Independent CRT | -0.110 (-0.23, 0.01) | 0.081 |
| One-to-One CRT | **0.157 (0.04, 0.27)** | **0.008** |
| Baseline GAS t-score | -0.028 (-0.14, 0.08) | 0.624 |

| Table S6: 6-month post-therapy SEM standardized structural coefficient estimates for the three models | | |
| --- | --- | --- |
|  | **Standardized Estimate (95% CI)** | **p-value** |
| Model 1 | | |
| 6-month post-therapy GAS t-score: |  |  |
| CRS score | 0.058 (-0.15, 0.26) | 0.577 |
| Baseline GAS t-score | 0.140 (-0.00, 0.28) | 0.055 |
| CRS score: |  |  |
| Baseline GAS t-score | 0.057 (-0.09, 0.21) | 0.474 |
| Model 2 | | |
| 6-month post-therapy GAS t-score: |  |  |
| CRS score | 0.044 (-0.16, 0.25) | 0.679 |
| Independent CRT | -0.138 (-0.31, 0.03) | 0.109 |
| One-to-One CRT | -0.023 (-0.18, 0.13) | 0.769 |
| Baseline GAS t-score | **0.15 (0.01, 0.29)** | **0.042** |
| CRS score: |  |  |
| Independent CRT | -0.097 (-0.29, 0.09) | 0.323 |
| One-to-One CRT | -0.019 (-0.19, 0.15) | 0.821 |
| Baseline GAS t-score | 0.060 (-0.09, 0.21) | 0.442 |
| Model 3 | | |
| 6-month post-therapy GAS t-score: |  |  |
| CRS score | 0.005 (-0.20, 0.21) | 0.964 |
| Hours of CR | 0.148 (-0.01, 0.30) | 0.058 |
| Baseline GAS t-score | **0.152 (0.01, 0.29)** | **0.032** |
| Independent CRT | -0.109 (-0.28, 0.06) | 0.217 |
| One-to-One CRT | -0.051 (-0.20, 0.10) | 0.510 |
| CRS score: |  |  |
| Independent CRT | -0.065 (-0.26, 0.13) | 0.518 |
| One-to-One CRT | 0.004 (-0.16, 0.17) | 0.965 |
| Baseline GAS t-score | 0.028 (-0.12, 0.18) | 0.716 |
| Hours of CR: |  |  |
| CRS score | **0.258 (0.10, 0.42)** | **0.002** |
| Independent CRT | **-0.160 (-0.27, -0.05)** | **0.006** |
| One-to-One CRT | **0.180 (0.06, 0.30)** | **0.002** |
| Baseline GAS t-score | -0.048 (-0.15, 0.06) | 0.379 |
